# Vascular risk factors for COVID-19 ARDS: endothelium, contact-kinin system

**DOI:** 10.3389/fmed.2023.1208866

**Published:** 2023-06-28

**Authors:** Melanie Bailey, Dermot Linden, Hong Guo-Parke, Olivia Earley, Tunde Peto, Danny F. McAuley, Clifford Taggart, Joseph Kidney

**Affiliations:** ^1^Mater Infirmorum Hospital, Belfast Health and Social Care Trust, Belfast, United Kingdom; ^2^Wellcome - Wolfson Institute for Experimental Medicine, Queen’s University Belfast, Belfast, United Kingdom

**Keywords:** bradykinin, inflammation, coagulation, endothelium, COVID-19

## Abstract

SARS-CoV-2 binds to ACE2 receptors, expressed within the lungs. Risk factors for hospitalization include hypertension, diabetes, ischaemic heart disease and obesity–conditions linked by the presence of endothelial pathology. Viral infection in this setting causes increased conversion of circulating Factor XII to its active form (FXIIa). This is the first step in the contact-kinin pathway, leading to synchronous activation of the intrinsic coagulation cascade and the plasma Kallikrein-Kinin system, resulting in clotting and inflammatory lung disease. Temporal trends are evident from blood results of hospitalized patients. In the first week of symptoms the activated partial thromboplastin time (APTT) is prolonged. This can occur when clotting factors are consumed as part of the contact (intrinsic) pathway. Platelet counts initially fall, reflecting their consumption in coagulation. Lymphopenia occurs after approximately 1 week, reflecting the emergence of a lymphocytic pneumonitis [COVID-19 acute respiratory distress syndrome (ARDS)]. Intrinsic coagulation also induces the contact-kinin pathway of inflammation. A major product of this pathway, bradykinin causes oedema with ground glass opacities (GGO) on imaging in early COVID-19. Bradykinin also causes release of the pleiotrophic cytokine IL-6, which causes lymphocyte recruitment. Thromobosis and lymphocytic pneumonitis are hallmark features of COVID-19 ARDS. In this review we examine the literature with particular reference to the contact-kinin pathway. Measurements of platelets, lymphocytes and APTT should be undertaken in severe infections to stratify for risk of developing ARDS.

## Introduction

Severe acute respiratory syndrome coronavirus 2 (SARS-CoV-2) has spread around the world rapidly. This novel beta coronavirus, responsible for coronavirus disease of 2019 (COVID-19) may result in a spectrum of illness ranging from asymptomatic infection to severe respiratory failure and death. COVID-19 is extremely contagious and is transmitted *via* respiratory droplets. Viral cell entry is enabled via the SARS-CoV-2 spike protein receptor binding domain (RBD) which attaches to the angiotensin-converting enzyme-2 (ACE2) receptor which is expressed in a wide variety of tissues, including pulmonary epithelial cells and the endothelium. With the help of transmembrane serine protease (TMPRSS2), the virus-receptor complex becomes internalized in cells enabling viral replication within the cell. The principal effects of initial infection are cough, fever, breathlessness, anosmia, loss of taste and infiltrates on the chest radiograph or computed tomography (CT). These infiltrates are alveolar filling defects, caused initially by oedema. A proportion of patients develop worsening symptoms and require hospitalization, often due to respiratory failure. These patients also develop coagulopathy, abnormal thrombosis, hypotension and acute renal impairment (1). Risk factors for progressive infection and mortality include age, hypertension, diabetes, ischaemic heart disease and obesity (2) suggesting that endothelial pathology may signify a unifying disease mechanism. Pre-existing endothelial dysfunction may cause excessive activation of the contact system in response to SARS-CoV-2 infection. The contact system is a key component of the innate immune response to inflammatory stimuli, tissue injury and infection. It is initiated by the conversion of the circulating zymogen Factor XII (FXII) to its active form FXIIa. This causes synchronous triggering of the intrinsic clotting pathway and the plasma Kallikrein-Kinin System (KKS). Activation of the intrinsic coagulation pathway causes clotting factor consumption (seen as raised APTT) and thrombus formation. Triggering of the KKS leads to the production of bradykinin, resulting in vascular proliferation, vasodilation, oedema, cough and IL-6 release (3). The diverse manifestations of COVID-19 infection could be due to sustained activation of these systems/pathways. Temporal changes in blood markers may reflect the actions of the contact-kinin system.

### The contact system and coagulopathy

FXII is a serine protease that circulates in plasma as a single-chain zymogen. After contact with anionic surfaces such as neutrophil extracellular traps (NETs), polyphosphates, activated platelets or DNA released from damaged cells, FXII undergoes autoactivation to FXIIa which triggers the intrinsic clotting cascade via FXI cleavage to generate FXIa.

**Figure 1:**
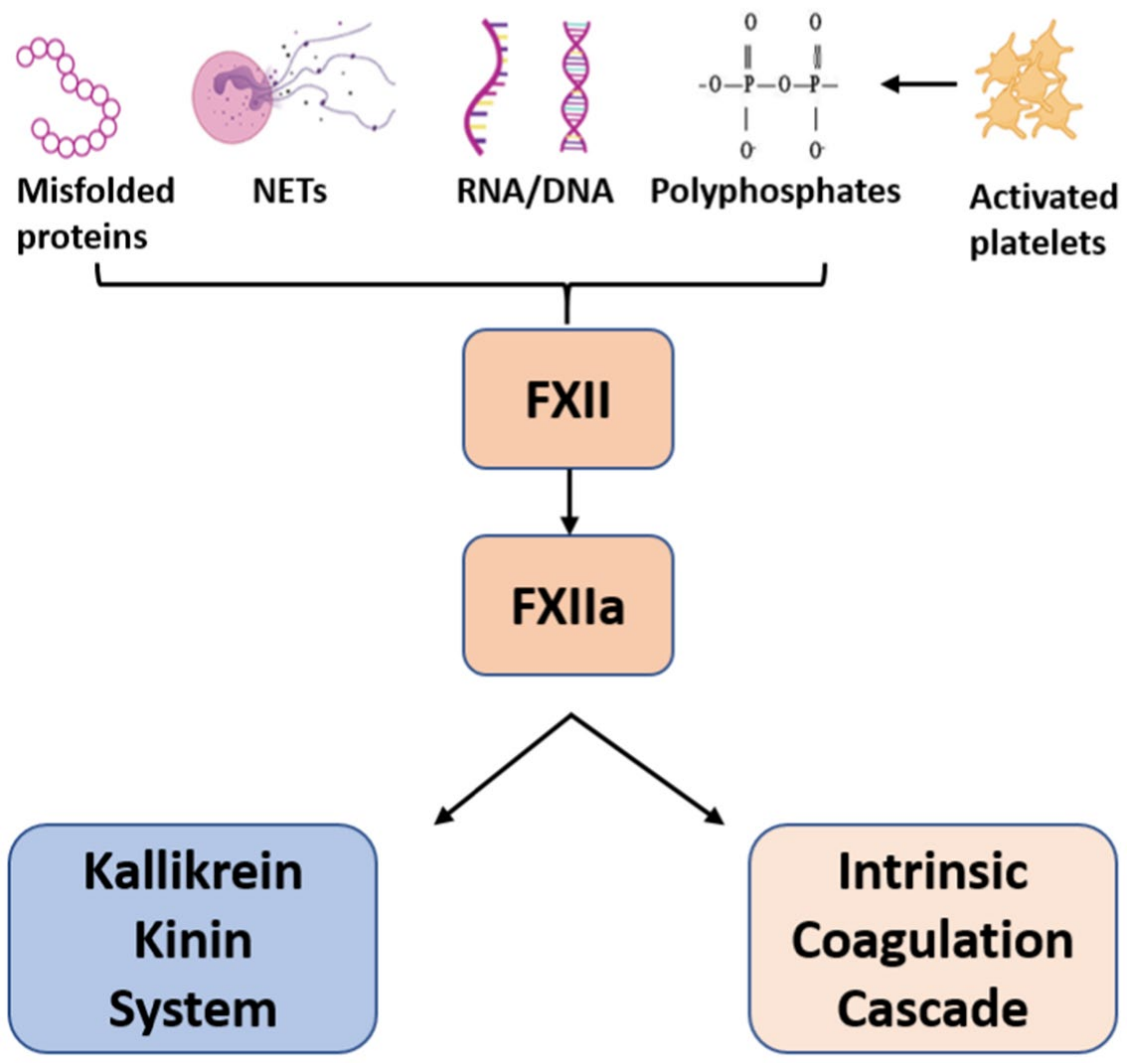
Activators of Factor XII. Factor XII is activated upon contact with anionic surfaces leading to dual activation of the Kallikrein-Kinin system and Intrinsic Coagulation cascade.

The subsequent proteolytic cleavage of factors IX and X ultimately leads to thrombin generation, fibrin formation and clotting (Figure 1). Activated partial thromboplastin time (APTT) measures the length of time taken for blood to clot via the intrinsic and common pathways. Prolonged results can indicate factor deficiencies/depletion or the presence of a competing antibody. APTT is often abnormally prolonged in COVID-19 (4). The mechanism behind this is not fully understood. Studies have identified the antiphospholipid antibody, lupus anticoagulant (LA) (5–7). Findings remain controversial as LA assays can be inaccurate in setting of raised C-reactive protein (CRP) or in patients on anticoagulant therapy (8) and these aspects were not always taken into consideration (9). Importantly, APTT prolongation is also found in LA negative COVID-19 patients (10–12). Prothrombin time (PT), fibrinogen and D-dimer levels are often abnormally elevated in COVID-19 (13, 14), and as the incidence of thromboembolism is known to be much higher than hemorrhagic events (15), APTT prolongation may therefore reflect clotting factor depletion.

### The contact system and kallikrein kinin system

**Figure 2:**
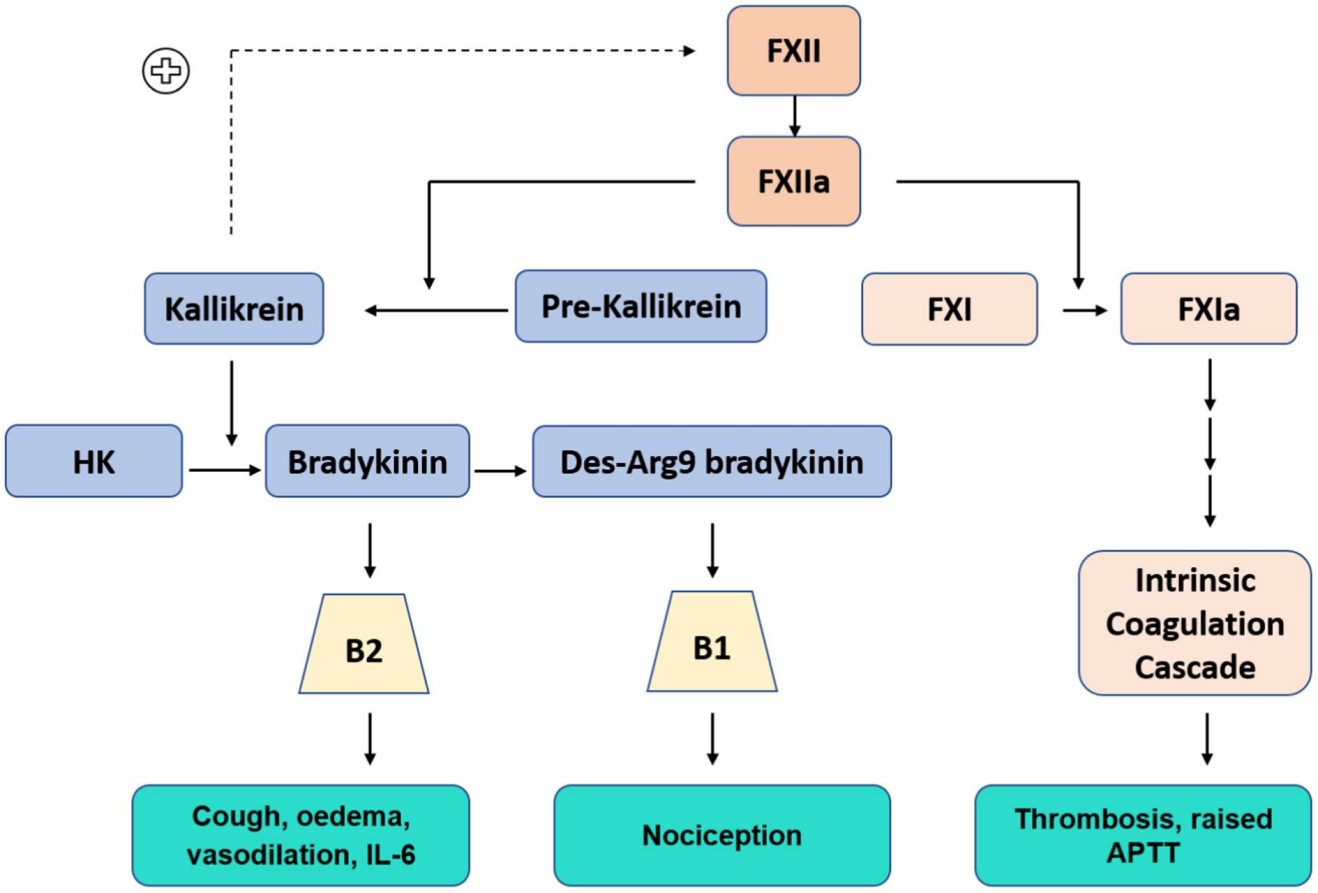
Contact Kinin System: Kallikrein-Kinin system (Blue) FXIIa cleaves pre-kallikrein to kallikrein (positive feedback loop to continue FXII activation), kallikrein cleaves high molecular weight kininogen (HK) to produce bradykinin, further processed to Des-Arg9 bradykinin. Bradykinin and Des-Arg9 bradykinin act at receptors B2 and B1, respectively. Intrinsic coagulation cascade (Orange)–abbreviated pathway.

In addition to triggering the clotting cascade, FXIIa also synchronously activates the KKS; a complex pathway that modulates inflammation, blood pressure control, coagulation and pain (Figure 2). This system is initiated when FXIIa cleaves plasma prekallikrein (PKa) to kallikrein, a serine protease which forms a feedback loop to continue the activation of FXII. Kallikrein then cleaves circulating high-molecular-weight-kininogen (HK), releasing bradykinin and subsequent derivatives. Bradykinin is broken down by ACE receptors. ACE inhibitor drugs are widely used for the treatment of hypertension, heart failure and diabetic nephropathy. Their effects are well established. Between 7 and 25% of people develop a dry cough (3, 16). A lymphocytic alveolitis can also occur (17).

Bradykinin binds to cell receptor B2, a G-protein-coupled receptor which is ubiquitously expressed in most human tissues (18). Further enzymatic processing of bradykinin produces des-Arg-9 bradykinin (DABK), a ligand of receptor B1, which is present on endothelial cells and upregulated in tissue injury and inflammatory states (19). Stimulation of these receptors has a potent effect; inducing vasodilation, vascular proliferation, micro-vascular permeability, oedema and release of pro-inflammatory cytokines including IL-6 and TNF-⍺ (20–22). Nagashima et al. studied lung tissue immunoexpression of B1 and B2 in post mortem lung samples from mechanically ventilated patients with COVID-19 (*n* = 24) and influenza (*n* = 10). Notably, expression of B1 and B2 were significantly increased in both COVID-19 and influenza patients compared to uninfected controls (*n* = 11) (23).

Bradykinin peptides can be generated independently of FXIIa due to the actions of proteases which trigger distal elements of the KKS. Prolylcarboxypeptidase (PRCP), a regulatory protease involved in the renin-angiotensin system (RAS) can cleave PKa to kallikrein (24). However, PRCP activity has been found to be similar in COVID-19 patients and controls (25). Cleavage of HK and kinin generation also occurs by agents other than kallikrein, such as the protease neutrophil elastase (NE), tryptase, cathepsins and proteinase-3 (PR3). These not only cleave HK but liberate bradykinin-like peptides with the ability to act at B2R (26, 27). Neutrophilia occurs frequently in COVID-19 and is associated with poorer outcomes (28). Neutrophils release a variety of potent enzymes including PR3 and NE (29). PR3 is a destructive protease with microbicidal activity and is capable of extracellular matrix degradation. Furthermore, PR3 can cleave high molecular weight kininogen producing PR3-kinin, which is then processed to bradykinin and des-Arg9-bradykinin in plasma (30). Increased PR3 has been reported in severe COVID-19 (31, 32). NE acts on elastin, collagens and HK - producing E-kinin, which is then cleaved to release bradykinin (26). Compared to healthy controls, progressively increasing NE levels correlated with hospitalization, need for ICU admission and mortality (33).

### Factor XII in COVID-19

Wygrecka et al. compared the levels of factor XII (FXII) and its activation products from critically ill COVID-19 patients with samples from influenza patients with severe ARDS (34). FXII was reduced, plasma kallikrein-like activity and HK cleavage products were increased in COVID-19 patients in comparison to influenza ARDS and controls. This shows the decrease in FXII is due to activation (34). A similar pattern of decreased FXII, PKa and HK was reported in a study of 66 intensive care COVID-19 patients (35). Neutrophil extracellular traps (NETS) have been implicated as triggers for the cascade in a study demonstrating the presence of FXIIa alongside NETS in lung parenchyma of COVID-19 patients (36). An increase in serum FXIIa was also seen in a separate group of COVID-19 patients (36). In a study of 128 COVID-19 and 16 healthy controls, admission FXII levels were increased in asymptomatic/ mild/ moderate disease in comparison to controls–possibly reflecting an acute phase response. However, FXII levels were significantly lower in those who progressed to severe disease (37). Importantly this finding is not limited to severely ill COVID-19 patients: reduced FXII levels have also been found in critically ill patients with sepsis (38), likely reflecting increased activation.

### Endothelial activity

Pre-existing endothelial pathology may be a priming factor for contact system activation in COVID-19. The endothelium is a complex organ that controls vascular tone, angiogenesis, clotting and the recruitment of leukocytes and platelets. These functions are regulated by expression of cell adhesion molecules such as E-selectin, intracellular adhesion molecule 1 (ICAM-1), vascular cell adhesion molecule 1 (VCAM-1) and thrombomodulin, an anticoagulant glycoprotein. Endothelial cells rapid responses are aided by organelles which function as storage granules called Weibel-Palade bodies. These store P-selectin, Von-Willebrand factor (vWF), eotaxin-3, interleukin-8, tissue-plasminogen activator, angiopoetin-2 (Ang-2), osteoprotegerin and endothelin-1. These are powerful inflammatory and physiological regulators which are rapidly expelled in response to vessel injury or inflammation. Vascular tone is regulated by endothelin-1, a potent vasoconstrictor. Angiopoietin-2, a form of growth factor, can function as a pro or anti-angiogenesis agent depending on inflammatory conditions and the presence or absence of other growth factors (39). P-selectin and vWF promote platelet and leucocyte adhesion. Activated platelets release adhesion molecules, perpetuating endothelial activation and cell recruitment. Von Willebrand factor polymerizes into large multimers that are size regulated by ADAMTS-13 (A Disintegrin And Metalloprotease with ThrombSpondin Motif type 1 motif, member 13), which is reduced in severely ill COVID-19 patients (40–42). This may reflect secondary consumption due to excessive release of vWF–which has been consistently shown to be markedly raised in the infection (43, 44). In a study examining components secreted from Weibel-palade bodies, blood from 39 patients with moderate/critical/fatal COVID-19 infection had significantly increased levels of plasma vWF, Ang-2 and osteoprotegerin compared to 15 healthy controls (45). When plasma was added to human endothelial cell cultures vWF increased significantly with critical/ fatal case plasma, though not with moderate disease plasma. Furthermore, plasma from fatal cases induced higher intracellular levels, but lower secreted levels of Ang-2, while inducing features of angiogenesis.

COVID-19 infection is also associated with elevated levels of endothelial P-selectin, E-selectin, VCAM-1 and Ang-2 (43, 46–49). At an early stage in the pandemic, Goshua et al. reported a significant increase in vWF factor and P-selectin in both non-ICU and ICU COVID-19 patients compared to controls (46). Notably, disease severity and mortality directly correlated with elevated vWF (46) and this trend has subsequently been shown in further studies (44, 50). Levels of soluble thrombomodulin (sTM) were found to have a mortality correlation (46). Philippe et al. studied results from patients with COVID-19 (*n* = 208) and unaffected controls (*n* = 29) (44). Ang-2, VCAM-1 and E-selectin were included in their analysis. Patients had bloods drawn on admission and were classified into groups (outpatient, non-critical and critical) according to outcomes over next 48 h. Compared to the control group, VCAM-1 was significantly increased in both the critical group and non-critical group. Elevated Ang-2, sTM and E-selectin was only demonstrated in the critical group. None of these markers varied significantly between the non-COVID-19 control group and the outpatient cohort of COVID-19 patients. A markedly different pattern was observed with vWF, with levels significantly elevated in all COVID-19 patients compared to controls. In addition, vWF levels further increased according to disease severity. Both of these results were seen in a smaller study of 50 mild/mod/severe patients and in a study of 28 patients (40, 51).

In a separate hospitalized group, E-selectin levels were raised in severe disease only, but Von Willebrand factor was raised in all patient groups with COVID-19. In 20 of the evaluated patients with mild/mod/severe disease, vWF levels remained elevated in 57% 30 days after admission (52). A larger study of 203 COVID-19 patients found that over 80% had raised vWF levels 3 months after the onset of infection (43). A study of 215 patients investigated endothelial markers in relation to transfer factor (DLCO) 6 months after COVID-19 illness. Patients with the lowest DLCO had higher levels of ICAM-1 and angiopoetin-2 (53). The prolonged lag in normalization may reflect the severity of the endothelial damage occurring during infection. Alternatively, it is possible that patients with persistent, abnormal endothelial markers may have had baseline abnormalities which accentuated their illness.

Viral infection causes oxidative stress and excess protein production in the endoplasmic reticulum. This overwhelms the regulatory unfolded protein response and results in loss of cellular homeostasis (54), which may accentuate the vigorous systemic response to COVID-19 in patients with pre-existing endothelial dysfunction. This includes the known high risk groups of older age, hypertension, ischemic heart disease, obesity, COPD and diabetes. For example, untreated hypertension has been associated with higher levels of vWF and P-selectin (55, 56). Levels improve with treatment though may still remain higher than control results (57). Other studies of hypertensive disorders have also found raised E-selectin (56, 58). Schumacher et al. found that patients with coronary artery disease (*n* = 193) have elevated levels of ICAM-1, VCAM-1 and P selectin compared to healthy controls (*n* = 193) (59). Patients with obesity have elevated levels of chemokines IL-6, TNF⍺ and MCP-1 and increased levels of endothelial ICAM-1, E-selectin, and P-selectin compared to those with a normal BMI (60). Elevated levels of vWF, E-selectin, ICAM-1 and VCAM-1 can be seen in patients with Type II diabetes–and can also precede diagnosis (61–63). Interestingly, increased vWF has also been found in patients experiencing acute COPD exacerbations (64). Hospital admissions due to COPD exacerbations are linked to a markedly increased risk of cardiovascular events (65). Polatli et al. compared levels of vWF in a healthy control group (*n* = 16), patients with stable COPD (n = 33) and patients with acute COPD exacerbations (*n* = 26). They found that the levels were progressively higher between the groups though not reaching statistical significance between stable/acute COPD (66).

### Histological features and ACE2

Post-mortem findings from patients with severe COVID-19 lung disease have shown marked endothelial dysfunction, lymphocytic infiltration, intussusceptive angiogenesis and widespread thrombosis with microangiopathy (67). Pathological abnormalities can also be seen in asymptomatic patients. Resected lung samples in patients who were subsequently found to have COVID-19 in the postoperative period have shown alveolar damage, oedema, proteinaceous exudates, vascular congestion, alveolar hemorrhage and interstitial inflammatory infiltrates (68–70). Histological abnormalities are not limited to the respiratory system, demonstrating the systemic nature of the infection. Lymphocytic infiltration, endotheliitis, oedema, microthrombi and vascular proliferation are found in organs such as small bowel, spleen, kidney and liver (67, 70, 71). As neurological symptoms and complications are commonly seen (confusion, delirium, headache, autonomic dysfunction, stroke and meningoencephalitis) histological examination of brain tissue is of significance in this systemic effect. There are ischemic lesions, oedema and microhaemorrhages. Lymphocytic infiltration is seen, affecting the brainstem in particular (72).

These findings are indicative of dysfunction at the epithelial/endothelial interface. Endothelial viral infection and endotheliitis in heart, lung, kidney and liver has been reported (73). A closer look at the mechanism of viral entry may provide an explanation for endothelial disruption. The ACE2 receptor serves as the binding site for SARS-CoV-2. Membrane bound ACE2 undergoes cleavage by ADAM Metallopeptidase Domain 17 (ADAM17) to form soluble ACE2, which plays a critical role in the renin-angiotensin system and the cleavage of numerous bioactive peptides including Des-Arg9-bradykinin (DABK). ACE2 is expressed in lung epithelial cells, the starting point of infection. It is known to be increased in the airways of COPD and current smokers (74). It is found throughout endothelial surfaces in veins, arteries and arterioles and also in arterial smooth muscle cells (75). However, an RNA sequencing study found that ACE2 expression on endothelial cells was low in comparison to epithelial cells from respiratory, gastrointestinal and skin sites (76). Similarly, early in the pandemic ACE2 expression was shown to be highest in small intestine, heart, kidney, adrenal glands, with blood vessels grouped among tissues with lowest expression (77). However, the endothelium is capable of mounting instant systemic responses and it is possible that low levels of expression are sufficient for damage. Bordoni et al. demonstrated that SARS-CoV-2 viral replication can occur in both airway epithelial and pulmonary endothelial cells (78). They also showed that culture of infected endothelial cells with non-infected pulmonary epithelial cells induced low levels of viral RNA in the pulmonary epithelial cells – though not vice versa. Moreover, viral infection of endothelial cells did not cause an increase in adhesion molecules (E-selectin, ICAM-1 and VCAM-1), but infection of epithelial cells resulted in increased E-selectin and VCAM-1, suggesting that the endothelial damage observed in COVID-19 is initiated by a cytotoxic effect from neighboring infected pulmonary epithelial cells.

Cells infected with SARS-CoV2 undergo pyroptosis leading to cytokines and danger associated molecular patterns (DAMPs). The response to these signals includes the release of neutrophil extracellular traps (NETS) and the activation of platelets. In addition to the damage caused by cell death, internalization of the viral/ACE2 complex leads to a relative ACE2 deficiency, which is significant, as ACE2 plays a vital role in the renin-angiotensin system by converting angiotensin II to angiotensin 1–7 (a vasodilator with anti-inflammatory, anti-thrombotic, antiproliferative and antifibrotic, activity) (79). The beneficial effects of ACE2 have been investigated in mouse models, showing that ACE2 deficient mice with acute lung injury improved following administration of recombinant ACE2 (80). Downregulation of the normal ACE2 function in COVID-19 leads to increased tissue and vessel exposure to angiotensin II, characterized by vasoconstriction, enhanced thrombosis, cell proliferation, increased tissue permeability, and cytokine production (81). Most importantly, a key agent of the contact-kinin pathway is bradykinin. ACE2 metabolizes des-Arg9-bradykinin.

### Hematological findings and dynamic changes

Blood results in hospitalized patients with COVID-19 often follow predictable trends, correlating with stage of infection. Blood markers can also indicate severity of disease and aid in predicting outcomes. Lymphopenia is regarded as a cardinal finding, occurring in approximately two-thirds of people with COVID-19 (82). The lowest values occur after 7 days from symptom onset (83), reflecting recruitment to affected tissues. Patients with more severe disease tend to have lower lymphocyte counts at point of admission and have a more prolonged period of lymphopenia than those with mild disease. A retrospective study described admission blood results and subsequent dynamic changes for 548 inpatients (84). In severe disease, average counts were lower than in patients with mild disease. Non-survivors display very low levels on admission without appreciable lymphocyte recovery (84, 85).

Neutrophils usually rise from infection onset (85). Neutrophilia is associated with disease severity and mortality (28). Throughout the pandemic, lymphocyte and neutrophil results have been combined to form the neutrophil/lymphocyte ratio. A score of >11.75 predictive of mortality in hospitalized patients (86). Evidence has suggested that neutrophils may promote organ injury and coagulopathy via direct tissue infiltration and subsequent formation of Neutrophil Extracellular Traps (NETs) (87). NETS are networks of extracellular fibers composed of DNA and histones complexes. Structurally, they play an important role in the entrapment of microbes. They are potent activators of FXII and thus mediators of thromboinflammation. There is evidence to suggest that neutrophils in COVID-19 patients produce higher levels of NETs and also longer complexes in comparison to those from healthy controls (88).

Platelet counts are also affected by COVID-19 infection. Thrombocytopenia is present on admission in over a third of patients (89) although it is typically mild. Studies correlating platelets with day of symptoms have shown that counts begin drop from infection onset and are markedly lower at 7 days post symptom onset. This is often overlooked as the reduction may still fall within normal parameters. The sudden decrease in platelets may reflect depletion due to activation and subsequent consumption. At a later stage in infection, a rebound increase occurs (90, 91)—often meeting criteria for thrombocytosis. This pattern was also observed in the 2002 SARS infection (92). Activated platelets are likely to be a major contributing factor to the disease pathology in COVID-19. Patients with critical disease have increased levels of soluble P-selectin and CD40 ligand (circulating markers of platelet activation) (93). The use of antiplatelet agents has been explored. Anti-platelet therapy has a number of potentially beneficial mechanisms, including inhibition of platelet aggregation, reduction of platelet activation inflammation, and blocking of neutrophil extracellular traps. The RECOVERY trial investigated the use of aspirin in hospitalized COVID-19 patients and found no difference in mortality compared to standard care (94). However, there may be a role for antiplatelet agents in the prevention of disease severity. A cohort study of 984 COVID-19 patients (253 pre-admission aspirin, 751 not on aspirin) reported that that requirement for respiratory support (as defined by non-invasive or invasive ventilation) was significantly lower in the pre-hospital aspirin group: 33% vs. 49%. They also found that this group had a lower neutrophil to lymphocyte ratio on admission (*p* = 0.013) (95). In addition, a large, propensity matched study found there was a 2.6% absolute reduction in mortality with pre-hospital antiplatelet agents (96).

There are limited studies reporting dynamic coagulation changes according to day of symptoms as the majority of studies report findings by day of admission. In a retrospective analysis of 843 COVID-19 inpatients the APTT was significantly higher after 5 days of symptoms, but not after 10 or 13 days (97). This is confirmed by a separate group which reported raised APTT levels from day 5 of symptoms in patients with severe disease which then decreased until day 10 (98). Similarly a significantly higher APTT level was seen in the first week of symptoms compared to the 4^th^ week (99). Studies reporting dynamic changes per day of admission have also demonstrated this trend. A meta-analysis of coagulation factors in COVID-19 reported that APTT levels decreased from point of admission, day 4 and onwards (100). A study of 1,131 hospitalized patients (36 severe) reported a slightly increasing trend in APTT and PT for the severe group and a slightly decreasing trend in the mild group within the first 10 days of admission (4). There was no temporal change in D-dimer for the mild group, but an increasing trend was observed in the severe group, a finding similar to a study of 320 hospitalized patients (101). Smadja et al. studied temporal trends of fibrin monomers, which are generated from the cleavage of fibrinogen from thrombin. They evaluated results from 246 inpatients during their first 9 days of hospitalization and demonstrated an increasing trend until approximately day 6 of admission in critically ill patients (102).

### Interleukin-6

In addition to abnormal hematological parameters, numerous pro-inflammatory cytokines have been detected and associated with the disease evolution of COVID-19. Studies of COVID-19 inpatients have revealed elevated levels of TNF-α, IFN-γ, IL-6, IL-8 and IL-10 (103–105). IL-6 is one of the most widely studied cytokines, leading to the utilization of targeted therapies such as tocilizumab and sarilumab. IL-6 is implicated in multiple processes, however it has a strong lymphotrophic effect, recruiting lymphocytes to the site of inflammation. Studies have consistently shown that elevated levels are predictive of disease severity and mortality (103, 104, 106). A cross sectional observation study of 272 COVID-19 inpatients demonstrated that a value of >79.9 pg./mL was predictive of unfavorable outcomes in terms of respiratory support and mortality (107). Santa Cruz et al. demonstrated that IL-6 levels peaked between 7 and 10 days post symptom onset in hospitalized patients (108). In keeping with risk factors for severe disease, IL-6 levels are higher in men and increase with age (105). This may be the link between high levels and severe lymphopenia and severe disease. It is notable that bradykinin causes release of IL-6.

### Radiological features

Chest imaging abnormalities are a signature feature of COVID-19 disease and may even be observed in asymptomatic individuals. Chest radiographs and CT imaging can demonstrate ground glass, oedema, consolidation and fibrotic change. For patients who require hospitalization, these changes also follow trends that can help to distinguish stage of disease. Many studies have correlated imaging findings with days of symptoms. Patients may have imaging changes without symptoms; a meta-analysis of 231 asymptomatic cases reported that 63% had findings on CT imaging–the majority were ground glass opacities (GGO) (109). Within the first 7 days from symptom onset, GGOs are the main radiographic finding (83) and are usually bilateral and favor mid/lower zones (110). After 7 days, consolidation increases and may lead to respiratory failure and ARDS in a proportion of patients. Management involves escalated therapy with ventilatory support *via* NIV/CPAP and may require treatment in intensive care with invasive ventilation, which is associated with a high mortality (111). A meta-analysis of risk factors for COVID-19 severity reported that male sex, hypertension, cardiovascular, cerebrovascular, diabetes and respiratory disease were significantly associated with progression to ARDS (112). Hospitalized patients have a high risk of venous thromboembolic disease. A meta-analysis of hospitalized patients reported a pooled incidence of pulmonary embolism in 14.7% of cases (113). Ackermann et al. carried out high resolution Syncroton imaging on lung samples from 31 COVID-19 patients who had died from respiratory failures, showing partial/total occlusion in sub segmental pulmonary arteries (114).

In addition to parenchymal changes and thrombosis, studies have reported enlargement/dilation of sub segmental blood vessels at an early stage of infection/ on admission to hospital (110, 115). Bianco et al. investigated this phenomenon in COVID-19 patients and compared the findings with CT scans carried out in patients with influenza pneumonia (116). They found that vascular enlargement was present in 90% (45/50) COVID-19 patients, but only seen in 24% (12/50) patients with influenza pneumonia. Poletti et al. carried out an analysis of the thoracic vasculature in COVID-19 patients (*n* = 279), Influenza patients (*n* = 159) and patients with normal CT chest imaging (*n* = 634). Blood vessel volume percentage (BV%) was calculated for small, medium and large vessels. In COVID-19, small vessel BV% were decreased in comparison to normal controls (14% vs. 18%), but larger vessels were significantly bigger (15% vs. 11%) (117). It is possible that these vascular changes develop due to dysregulated release of bradykinin and alterations in the RAS as a consequence of ACE2 downregulation.

### Inhibitors of the contact system

The contact system is known to be regulated by several physiological inhibitors. C1-esterase inhibitor (C1-INH) is the main inhibitor of FXIIa and FXIa and also acts to inhibit plasma kallikrein and the complement classical and lectin pathways. C1 inhibitor deficiency is the main cause for hereditary episodic angioedema (HAE); a rare genetic condition characterized by bradykinin mediated episodes of oedema. Consequently, individuals with C1-INH HAE have reduced regulation of the coagulation system and are at an increased risk of thromboembolic disease (118)—a finding recently replicated in a mouse model study (119). There have been differing reports of C1-INH in COVID-19. At the start of the pandemic, a study of admission blood results from 154 COVID-19 inpatients reported elevated levels of C1-INH which correlated positively with peak CRP, LDH and ferritin (120). A smaller study also reported increased C1-INH levels in 5 patients with severe disease (121). Conversely, 27 COVID-19 positive hemodialysis (HD) patients compared with 32 COVID-19 negative HD patients had significantly lower levels of CI-INH in the positive group. However, both HD groups were elevated in comparison to healthy controls (122). Other agents that can inhibit FXIIa (to a lesser extent) include antithrombin, α2-antiplasmin and α2-macroglobulin (123). Antithrombin targets FXIIa, FXIa and plasma kallikrein. Several studies have identified lower antithrombin levels in COVID-19 non-survivors compared to surviving groups (123–126). α2-antiplasmin is the main inhibitor of plasmin and can also inhibit FXIIa. Data on α2-antiplasmin levels in COVID-19 is limited, however similar results have been found between healthy control groups and admission values for COVID-19 patients (127, 128). α2-macroglobulin regulates several proteases including kallikrein and FXIIa. De Laat-Kreemers et al. reported that α2-macroglobulin levels were within normal range in 133 COVID-19 inpatients, but significantly lower levels were noted in patients with thrombosis compared to those without (129).

### C1 esterase inhibitor

C1 esterase inhibitor - inhibitor of FXIIa and FXIa and also acts to inhibit plasma kallikrein and the complement classical and lectin pathways. A small case–control study compared the outcomes of 5 COVID-19 patients who received C1-esterase inhibitor with 15 matched controls. The baseline characteristics and admission laboratory parameters were similar between groups. Eight patients (53%) in the matched control group died or required mechanical ventilation compared to only 1 (20%) in the C1-esterase inhibitor group (121). Another study reported the results of a randomized, open label trial of C1-esterase inhibitor, bradykinin B2 receptor antagonist (icatibant) or standard care alone (*n* = 9, 10, 9 respectively) (130). COVID-19 patients with severe infection (radiological pneumonia; SpO_2_ ≤ 94% in ambient air or Pa0_2_/FiO_2_ ≤ 300 mmHg) were recruited and allocated to treatment arms 1:1:1 up to day 12 of symptoms. There were no significant differences between groups in terms of time to clinical improvement or mortality. There was an improvement in lung CT scores. The size of this study would require a strong signal to identify clinical improvement or mortality.

### Plasma kallikrein inhibitors

Plasma kallikrein inhibitors have been developed for use in hereditary episodic angioedema (HAE). Lanadelumab and berotralstat are licensed for the prevention of recurrent attacks. Lanadelumab is administered as a subcutaneous injection every 2–4 weeks, depending on disease stability. Trials in COVID-19 are currently underway. Berotralstat is taken orally once per day. Both medications have been shown to significantly reduce the incidence of attacks compared to placebo (131, 132). Ecallantide is a plasma kallikrein inhibitor used to treat acute HAE attacks (133). Donidalorsen is an antisense oligonucleotide treatment which results in decreased production of PK from the liver. A phase 2 study has demonstrated a reduced rate of angioedema attacks compared to placebo (*p* = <0.001) (134).

### Factor XII/FXIIa inhibitors

Garadacimab, a novel FXIIa inhibitor has been recently trialed in a phase 1 study (135). Dose-dependent increases in plasma concentration and pharmacodynamic effects in kinin and coagulation pathways were observed. Trials are currently underway in COVID-19.

### B2 receptor blockade in COVID-19

Bradykinin receptor activation may cause the radiological oedema, hypotension and cytokine release seen in early COVID-19 infection. The use of a receptor antagonist may interrupt this pathway and prevent progression to secondary damage, as defined by inflammatory cell infiltrates, thrombosis and vascular injury. Icatibant is a B2 receptor antagonist that has been in use since 2008 for the treatment of oedema caused by hereditary angioedema. It has a rapid onset of action, with median time of 2–2.5 h to symptom relief following a single dose (136, 137). It has a short half-life of 1.48 +/− 0.35 h (138). Published data on the use of icatibant in COVID-19 is limited to four studies and two case reports. Importantly, there have been no safety concerns noted regarding the use of icatibant in COVID-19 in existing reports.

A case report of a single patient with severe COVID-19 infection described a favorable clinical response after icatibant (139). Giol et al. reported radiological improvement in a COVID-19 patient who received icatibant as a treatment for ACE inhibitor induced angioedema (140). Van de Veerdonk et al. described decreased oxygen requirements in COVID-19 patients (*n* = 9) treated with open label icatibant compared to standard care (*n* = 18). After 24 h, 89% of patients receiving Icatibant had a reduction of at least 3 L of oxygen compared to only 17% of controls (141). Mansour et al. carried out a randomized, open-label trial with three treatment arms; icatibant, C1 esterase inhibitor and no trial drug (“usual care”) (130). Eligible patients had hypoxia, radiological COVID-19 pneumonia and were within 12 days of symptom onset. Patients in the icatibant arm received 30 mg three times per day for 4 days. The C1 esterase group were administered a dose on days 1 and 4. The main outcomes were time to clinical improvement as defined by the WHO ordinal scale and improvement in lung CT scoring. There were no significant differences between the groups.

Malchair et al. carried out a randomized, open label trial of icatibant 30 mg three times per day for 3 days plus standard care compared to standard care alone. Patients were eligible if requiring supplemental oxygen, but not high flow oxygen or ventilation. Patients were required to have a positive PCR or antigen test within 10 days of randomization although symptom onset was not recorded. The primary outcome was clinical response (defined as category 2 or lower of the WHO ordinal scale, sustained for 48 h) by day 10 or discharge. The primary outcome was assessed for 37 and 36 patients, respectively. 73% of patients in the icatibant arm (27/37) and 53% of standard care patients (20/36) met the clinical response target, although this did not meet statistical significance. However, clinical response was maintained at day 28 by all of the patients in the icatibant arm compared to controls (*p* = 0.011) and icatibant patients had a significantly shorter inpatient admission, 8 days vs. 10 (*p* = 0.014) (142).

A platform study has recently published data for the first seven medications trialed (including icatibant). The trial recruited patients with severe/ critical disease (requiring ≥6 L of oxygen) and an average symptom history of 9–10 days. There were 96 patients enrolled in the icatibant arm. Over half of the patients required ≥15 L of oxygen and a third were on non-invasive or mechanical ventilation. The primary aim was to find a large signal in terms of improved recovery time and mortality. This was not achieved for any of the medications used (143).

## Conclusion

Activation of the bradykinin pathway begins at infection onset. In a proportion of cases this process eventually leads to inflammatory cell infiltration and tissue damage. These features are most evident at a later stage of the illness (>7 days post symptom onset). At this point, lymphocyte counts are lowest and neutrophil counts increase. Chest radiological appearances demonstrate progression from initial ground glass appearance (oedema) to dense consolidation. It is acknowledged that widespread vaccine uptake and the emergence of other variants are associated with milder illness in COVID-19. However, patients with vascular co-morbidities remain at risk of infection progression and this may be due to accentuation of the contact-kinin system in setting of endothelial dysfunction.

The study authors are conducting a proof of concept trial of icatibant in hospitalized patients with early COVID-19 infection. The aim is to trial bradykinin blockade in early, potentially reversible disease, before the onset of secondary damage and acute respiratory distress syndrome (ARDS). Patients who have had symptoms for more than 7 days will not be included. The primary outcome is to determine the effect on the Alveolar-arterial gradient (as measured by arterial blood gas before and after treatment). If an improvement in oxygenation is found, icatibant or alterative medications targeting the KKS could be tested on a larger scale. This could potentially change the management at point of admission for early COVID-19, preventing progression to respiratory failure. There may be wider implications for lung injury, as a study of bradykinin inhibition showed an improvement in the 28-day risk-adjusted survival in patients with SIRS from gram-negative infections (144).

## Author contributions

All authors listed have made a substantial, direct, and intellectual contribution to the work and approved it for publication.

## Funding

MB is funded by the R&D office for an NIHR adopted COVID-19 study (Grant number: COM/5612/20).

## Conflict of interest

The authors declare that the research was conducted in the absence of any commercial or financial relationships that could be construed as a potential conflict of interest.

## Publisher’s note

All claims expressed in this article are solely those of the authors and do not necessarily represent those of their affiliated organizations, or those of the publisher, the editors and the reviewers. Any product that may be evaluated in this article, or claim that may be made by its manufacturer, is not guaranteed or endorsed by the publisher.
